# Planning for translational research in genomics

**DOI:** 10.1186/gm87

**Published:** 2009-09-29

**Authors:** Naomi Hawkins, Jantina de Vries, Paula Boddington, Jane Kaye, Catherine Heeney

**Affiliations:** 1 The Ethox Centre, Department of Public Health, University of Oxford, Old Road Campus, Headington, Oxford OX3 7LF, UK

## Abstract

Translation of research findings into clinical practice is an important aspect of medical progress. Even for the early stages of genomics, research aiming to deepen understandings of underlying mechanisms of disease, questions about the ways in which such research ultimately can be useful in medical treatment and public health are of key importance. Whilst some research data may not apparently lend themselves to immediate clinical benefit, being aware of the issues surrounding translation at an early stage can enhance the delivery of the research to the clinic if a medical application is later found. When simple steps are taken during initial project planning, the pathways towards the translation of genomic research findings can be managed to optimize long-term benefits to health. This piece discusses the key areas of collaboration agreements, distribution of revenues and recruitment and sample collection that are increasingly important to successful translational research in genomics.

## Correspondence

The translation of research findings into clinical practice is an important aspect of medical progress. Even for the early stages of genomics research, which aims to deepen our understanding of underlying mechanisms of disease, questions about the ways in which such research ultimately can be useful in medical treatment and public health are of key importance. The aim of this paper is to put forward concrete ways to enhance the process of translation by focusing on the key considerations that need to be taken into account in the early planning stages of a research project when the translation of research findings into clinical use may seem quite remote. Whilst some research data may not apparently lend themselves to an immediate clinical benefit, being aware of the issues surrounding translation at an early stage can enhance the delivery of the research to the clinic if a medical application is later found. When simple steps are taken during initial project planning, the pathways towards the translation of genomic research findings can be managed to optimize long-term benefits to health. This paper discusses the key areas of collaboration agreements, distribution of revenues, and recruitment and sample collection that are increasingly important to successful translational research in genomics. Such consideration is timely in light of the recent report on Genomic Medicine by the House of Lords in the UK, which recognized translation as vital to realizing the potential of genomic medicine, and the need to address various obstacles to successful translation [1].

## What does translation mean in terms of genomics?

A significant proportion of genomics research is still at a very early stage in terms of clinical outcomes. Despite early excitement about the results of genome-wide association studies, recent debates in the scientific literature highlight that most of the variants found through this methodology account for only a small degree of the relative risk of developing a disease or a trait, and the findings collectively explain only a very small proportion of the underlying genetic component of most diseases [2,3]. Critics of current methodologies advocate increased focus on the study of rare variants and gene-gene and gene-environment interactions [4,5]. Whilst there may be debates about the appropriate allocation of funding to different research methodologies within genomics research, it is clear that genomics research will continue to advance rapidly.

Although there is uncertainty as to the actual concrete benefits that are likely to accrue from genomics research [6-9], there seem to be three major areas into which clinical outcomes of genomics research could be grouped. Firstly, the research may lead to better understanding of physiology and disease states [10]. It is hoped that these advances will influence and improve current treatment practices. For example, a recent paper outlines the developments in the understanding of the genetic architectures of plasma lipids and lipoproteins, anticipating that these advances may improve classification, diagnosis and treatment of dyslipidemias [11]. Secondly, the research may lead to the development of new or better diagnostic tools - for example, for disease stratification or predictive tests for common complex diseases [3]. It is hoped that the development of such tests may contribute to early diagnosis, screening programs or strategies that delay or prevent the onset of disease. Genetic tests exist for many rare genetic disorders, including cystic fibrosis and hypertrophic cardiomyopathy. Early predictive tests or useful diagnostic tests may also be developed for common complex disorders in the future. Moreover, pharmacogenetic tests are also in development (for example, recent research has identified genetic variants linked to susceptibility to developing statin-induced myopathy [12]) and some are already in clinical use [13] (for example, in the prescribing of abacavir for the treatment of HIV [14]). Commentary on recent efforts to translate the results of genome-wide association studies into direct-to-consumer tests has indicated the difficulties in developing tests proven to have clinical validity and utility, and regulating their sale [15-17]. Third, genomics research may lead to the development of new therapeutics, although there has not been the rapid discovery of novel and perfect drug targets that was anticipated by some when the human genome was first sequenced [18].

Much recent commentary has focused on the achievements and limitations of genomics research to date [19-21], and on further directions to advance science and maximize clinical utility [22,23]. This paper aims to contribute to this endeavor by examining how initial steps can maximize the utility of this immense research effort.

## Translation via commercialization

Commercialization is an important means by which innovations in biomedicine are translated into clinical practice, and whilst the positive and negative aspects of industry involvement in biomedicine are still hotly debated, commercialization is a reality in translational research [24]. The development of clinical products for patients usually occurs with the involvement of industry, for the oft-cited reason that the regulatory approval processes to which biomedical products are subjected require significant time and money [25,26]. In genomics, the importance of industry in translation is likely to vary with the difficulty and length of development of a product following a basic science discovery, as well as the degree of regulatory oversight and approval of the product in question.

There are increasingly permeable boundaries between basic and applied, academic and industrial science. Many genomics research projects are now set up with a commercial partner (for example, Procardis [27]); some European Union projects call for the involvement of small and medium enterprises [28], or a commercial partner may become involved later in the life of a project [29]. The necessity of effective collaboration between industry, academia, the charitable sector and the UK National Health Service for translational research was highlighted in the recent UK House of Lords Genomic Medicine Report [1]. Factors such as the reputedly high revenues gained by public research institutions following the patenting of new inventions, and institutional policies and pressures have meant that many public science researchers are encouraged to patent and develop their research findings [30-32]. However, it remains the case that later stage development of innovations is conducted primarily by or in partnership with companies.

Given these realities of commercialization, there are a number of issues that should be considered early in a genomics research program. In this paper we discuss the types of agreements that must be put in place, the factors that funders and research institutions need to consider and the importance of informing participants to ensure public trust in research. This process does not need to be costly or time consuming. Moreover, consideration and appropriate attention at an early stage may save time and money later on.

## Setting the ground rules: collaboration agreements

Genomics research frequently involves collaborations between many researchers from different institutions in multiple countries. Research collaborations are forged between researchers who have different but complementary expertise, skills and resources. These collaborations are often based on informal relationships; previous experience of working on research projects together or on the reputation that an individual or group may have gained in the field.

However, once funding is obtained for such a project, formal agreements will be put in place between the researchers, funders and institutions involved in the research. The primary aim of such contracts is to articulate the nature and aims of the project and the roles and responsibilities of each party, but they do not necessarily articulate the fine detail of all aspects of the project. They also will need to cover, amongst other things, issues such as ownership of intellectual property (IP) - including defining the ownership of prior held IP and that developed during the project - confidentiality and material transfer.

Other aspects that might benefit from early consideration and articulation include rights of research and experimental use, and publication and dissemination rights. Overly restrictive agreements that constrain future research direction and prevent researchers developing and pursuing autonomous research are rarely appropriate or warranted for academic research. Some delay in publication for the purposes of obtaining IP protection may be necessary, but the delay should not be protracted. Policies and best practices for university licensing have been developed that emphasize reservation of research rights and licensing in a way to maximize use and development of inventions, and may provide useful guidance [33-35].

Putting these agreements in place is not straightforward. Each of these contracts raises different issues for the parties involved and can be time-consuming to negotiate and develop. Moreover, where projects receive funding from multiple sources and involve multiple parties, sometimes in many different countries, each with different legal regimes, the challenges are magnified. Additionally, it can be exceptionally difficult to draft agreements that both accommodate the current organization of the research and are also flexible and provide appropriate solutions for future possibilities. Model agreements have been developed to assist parties and these could be a useful starting point [36,37]. Efforts in some jurisdictions to standardize agreements have made the process of developing these agreements easier, but this has not happened in all jurisdictions. Especially in complex projects spanning multiple institutions and jurisdictions, agreements need to be tailored to the specific situation [38]. Moreover, agreements need to be drafted in a manner sensible and sensitive to downstream development. An overly restrictive agreement put in place at an early stage, although conceived with the best of intentions, may in effect rule out any development if it includes conditions that are extremely difficult or impossible to fulfill for potential commercial partners.

Whilst there may be increased transaction costs associated with consideration of these issues at an early stage, these are outweighed by two advantages of having clear agreements in place before the commencement of research. Firstly, the process of drafting these agreements means that full consideration is given to the issues, which can mean that potential pitfalls are recognized and avoided. Secondly, if disputes do later arise, the agreements will hopefully provide a basis and mechanism for their resolution.

These agreements are essential. Although precise details of problems are often kept confidential due to their commercial sensitivity, insufficient attention to detail in this area has led to significant stumbling blocks for potentially valuable projects, sometimes in the initial stages of getting a project off the ground, whereas for others problems with agreements have not become apparent until later in the research project.

## Distribution of revenues

Research institutions, funding bodies and individual researchers may all have an interest in protecting possibly profitable outcomes from the work that they fund, host, or execute. Increasingly in recent years, universities have seen the commercialization of research as a potential source of income and as a means to fund further research. Similarly, funding bodies may have a direct interest in promoting the commercialization of the research that they fund, as this may generate revenue for the future [39].

Whilst there is the potential for 'blockbuster' revenues from patents in genomics, such as the benefit Stanford University and the University of California gained in terms of additional research funding from the licensing revenues of the Cohen-Boyer patent [40], this is exceptional. Patents are expensive to acquire and maintain, and costs may not match up to the benefits. In addition, the patenting landscape in genomics is complex, with the potential for multiple patents covering genetic sequences or variants, methods and techniques. The development of a genetic diagnostic test for a common complex disorder may therefore require the careful negotiation of licensing agreements with multiple stakeholders, each of whom will seek a percentage of revenue as a royalty. Huge license fees are not likely to be workable in relation to patents that make a small contribution to a larger product. It is unrealistic in such a circumstance to expect and bargain on the basis of wanting a huge percentage of profit; to do so may ultimately block translation [41].

Participants are an essential partner in genomics research projects; research cannot proceed without access to samples. This fact necessarily raises the question of whether the donors of the genetic material have a right to compensation or reward; so-called 'benefit sharing' [42]. This compensation, whether financial or non-financial, might be at the time of donation of the sample, or at some future point in time, if there are ultimately profits generated from the research project [43,44]. Some projects might provide for individual benefits, such as the feedback of clinically relevant results such as blood pressure from initial assessments or medical treatment [45,46]. Others might aim to provide community benefits through initiatives such as building schools [47]. The question of sharing of benefits is complex, and a full consideration is beyond the scope of this paper. We agree with Forsberg *et al. *[48] that research is necessary and desirable for future development of healthcare, and excessive focus on individual benefits at the expense of solidarity and altruism may provide a barrier to research. One way to consider benefit sharing in genomic research is to focus less on individual benefits, and more on benefits for public health [48].

One aspect of benefit sharing in the literature is the question of ensuring fair and reasonable access to the innovation for those who participated in research to develop it. Access is important not only for those who directly participated in the research in question through donation of samples, but also for the population as a whole; innovations of genomics research should be available to those who need them, not only the privileged few. Questions of access to healthcare are not specific to genomics; they take place within a much broader context of healthcare and social justice and equality.

The general assumption is that in a system of social healthcare, there is automatic access to the medical innovations through regular healthcare provision. However, with increasing financial pressures on health services, it may not in fact be reasonable to assume that genomic innovations will be standard of care, especially where such innovations are very expensive, such as in the case of Herceptin in the UK. If a social healthcare system does not pay for the innovation in question for those whose genetic information helped to develop the innovation, does the commercial developer have a duty to do so? In some cases, this may be reasonable. But where an innovation was developed for a very small population, where a large proportion of them were involved in the genomic research, then a requirement that the innovation be provided free of charge may mean that there is effectively no market for a product. In such circumstances charitable and government funding, as well as tax concessions, may be helpful to make development possible.

Both universities and funding bodies may be in a better position than individual researchers to use their considerable negotiating power and access to legal advice to develop frameworks for translation of their funded research that will maximize patient access. Funding bodies such as the Gates Foundation, whose prime concern is ensuring the equitable distribution to vulnerable populations of innovations developed through funded research, use IP rights and contractual means to ensure this. For example, researchers seeking access to data generated by the MalariaGEN project are required to sign up to an agreement that includes the following: 'if the use of the data gives rise to IP that could support health benefits in the developing world, the owner of the IP agrees to license it on a reasonable basis for use in the developing world and on a preferential basis to the countries whose citizens are the subject of the database' [49]. What remains undefined, however, is what 'licensing on a reasonable basis' means. Others who promote 'global social responsibility' propose concrete licensing models for university inventions that aim to improve access for the developing world [50]. Vigilant attention to the actual impact of these license agreements is necessary to ensure they are reasonable, flexible and assist in providing effective sustainable translation of genomic research.

## Recruitment and sample collection: consent and commerce

The expected benefit to patients from genomics research is a major motivating factor for participants in genomics research; even if participants have no expectation of personal benefit, they are generally motivated by potential improvements in the health of the population [51]. At the same time, participants often have reservations about commercial involvement in, and profit from, research on their samples and information, as well as concerns about who will benefit. Beliefs about the potential for research to give rise to improvements in healthcare may result in tension if coupled with misconceptions about what this would involve. If these tensions are not appropriately managed, this could be problematic for the sustainable translation of research results in genomic research that relies on the continuous recruitment of patients and controls. However, simple steps could help to sustain recruitment and enhance public trust in the research and translation process.

Amongst potential research participants, there is a wide range of opinion opposing or supporting commercial involvement in genomic research. Those who question the extent of commercial involvement in research may express concerns about industry profiting from what they see as 'their' biological material [52]. Groups such as Genewatch UK express concerns about the costs of products, or that commercial involvement may skew research agendas towards commercially viable products at the expense of other outcomes that may be of importance [53]. Meanwhile, the involvement of commercial partners in research may be explicitly promoted by some patient groups, such as PXE International, that actively seek out and encourage industry to investigate and develop therapies for their diseases [54]. The Genetic Interest Group, an umbrella organization representing the interests of those affected by various genetic diseases, both receives funding from industry and actively participates in research, often with commercial partners and with the explicit aim of producing outputs such as pharmacological treatments [55]. Others may even be prepared to pay to be involved in commercial genomics research projects [56].

### Informing participants

Consent forms may vary in the information they provide. Where research explicitly involves a commercial partner, it is likely that the consent process will have taken the possibility of patenting and financial profit into account. Participants will then have been informed of commercial involvement before consenting. Difficulties may arise where archived data are used or where data are shared from studies taking place within a purely academic context. Consent information from such studies may refer to possible sharing of data or their use in other 'research' or 'scientific research' or 'medical research', but rarely mentions industry. Hence, participants may not be aware of the possibility of downstream involvement of industry, private profit and patenting.

The context of consent is well known to be of crucial importance to shaping participants' expectations and understandings of what research will involve. Many taking part in clinical research still partake of the 'therapeutic misconception' that they may benefit from research, even if it has been explained to them that benefit is unlikely [57]. Likewise, research taking place within a purely academic context, such as a research institute or hospital, especially where publicly funded, is likely to lead at least some participants to think of the research as basic research rather than research leading to possible commercial activity [58]. Researchers cannot assume that participants will realize that the translation of research into concrete medical outcomes generally involves commercial partners with possible profits for some.

Moreover, research indicates that commercial involvement is something recruits wish to know about, even if it will not change their decision to participate [59-61]. Research also tells us that fostering trust between potential participants and researchers holds the key to sustainable recruitment practices [51,60,62]. Being explicit about downstream commercial involvement may assist in fostering such a climate of openness and trust. It also means that consent is obtained for future commercial collaboration without the need for re-consent on this issue.

### Consent and commerce: what are recruits told?

Many studies taking place within an academic context understandably make no reference to the issue of the translation of research findings. For example, the highly respected Whitehall studies, run from University College, London, and currently funded by the Medical Research Council, British Heart Foundation, the National Heart Lung and Blood Institute and the National Institute of Ageing, have been taking place over several decades within different levels of the UK civil service. These studies have produced voluminous and much utilized research findings, including data that have been used in genomic research. The consent form for the latest phase of the project (the Whitehall Phase 9 Consent Form [63]) does not make explicit mention of whether or not there is any commercial involvement, as the following extract indicates:

*'I consent to participate in the genetic component of the Stress and Health Study. DNA will be prepared from my blood cells for the study of genetic influences which may be relevant to diabetes, heart disease, stroke and cognitive function, and their risk factors. DNA will be stored for use in projects undertaken by the Stress and Health Study and its collaborators. I understand that no information found from the DNA will be given to me and that the information will be treated in the strictest confidence. I understand that the samples and information will be coded and used anonymously for research purposes only*.

17. I agree that the blood samples that I have given and the information gathered about me will be stored by the Stress and Health Study for possible use in future research projects. Samples and information will be anonymised, so that I will not be identified, before being used in future research projects. All my personal information will be treated in the strictest confidence in accordance with the Data Protection Act (1998) and samples in accordance with the Human Tissue Act (2004).'

This consent form makes it clear that samples and data may be used in future research projects, and as such leaves open the possibility that such future research projects may lead to translational outputs with commercial components. However, where a research project may share data in future research projects with research groups involving commercial partners, it may be advisable specifically to mention the possibility of this and of outputs with financial rewards for some parties.

The Procardis Programme aims to discover novel susceptibility genes for coronary artery disease and involves collaboration between various European universities as well as commercial partners [27]. Therefore, the consent form explicitly mentions the element of commercial partners and future translation of research:

'I understand that the University of Oxford, and its academic and commercial partners in the study, will use the results to try to improve the diagnosis and treatment of patients (including, for example, patenting and developing new drugs), and that I shall not benefit financially from my participation.'

Other projects that contemplate data sharing may specifically address the question of commercial outcomes in their consent forms. For example, the model consent form developed for the National Human Genome Research Institute Medical Sequencing Project [64] specifically addresses this question:

'3. Financial Compensation/Costs

You will not be paid to participate in this project. Your blood (or other tissue) samples and your medical information will be used only for research purposes and will not be sold. It is possible that some of the research conducted using your samples or information eventually will lead to the development of new diagnostic tests, new drugs or other commercial products. Should this occur, there is no plan to provide you with any part of the profits generated from such products.'

The Kadoorie Study of Chronic Disease in China is a collaborative, longitudinal study between the Clinical Trials Service Unit at the University of Oxford and the Chinese National Centre for Disease Control involving 500,000 recruits. Data recorded include lifestyle data, such as smoking, and several clinical measures, such as blood pressure and lung function, and a blood sample is taken. Recruits are to be monitored for 10 to 20 years. This project has explicitly ruled out commercial involvement. The consent form for this project (available on application [65]) specifies:

'I understand that all information provided by me will be treated confidentially, and that the Chinese National Centre for Disease Control and the University of Oxford that are responsible for the whole project will use the results to try to improve the prevention and treatment of common disease, and that I shall not benefit financially from my participation. The information and blood samples collected will not be used for any commercial purposes.'

Such a statement is open to interpretation in different ways. Especially given that the results of such a study can be expected to have widespread significance for populations worldwide [66], it seems highly likely that the results will then feed into understandings of disease that help ground the development of commercial products. Close attention to what is being specified in consent forms about commercial use of participants' data and samples, and end results, is advisable. Whilst researchers collecting samples and performing initial analysis of data may have no intention of commercial activity, data sharing practices complicate promises that there will be no commercial involvement.

P^3^G (the Public Population Project in Genomics) is an international consortium to promote collaboration between researchers in the field of population genomics that develops tools to facilitate data sharing in genomics. It has developed a generic consent form [67] for adaptation for research projects that includes a disclaimer about commercial involvement in research:

***'*** *COMMERCIALIZATION*

I understand that with proper oversight, results and samples may be exchanged with researchers in other countries, including those from commercial companies, for use in specific biomedical projects. I will not receive any personal financial benefit from the commercialization of any test or product that may result.'

A clear recommendation is that the simplest, most direct and most honest approach is to indicate explicitly the possibility of commercial involvement in any consent forms and background study information, where there is any chance that research data might be used later on for such purposes. The use of archived data for translational research is likely to be consistent with original consent so long as commercial involvement was not explicitly ruled out. However, for current research, best practice is to be explicit about the possibility of commercial outputs. This would help in countering the possible misconception whereby some recruits may distinguish between 'pure' research taking place in a university or other academic context, and research with links to commercial outcomes [68]. It is also in line with findings about information that recruits would wish to be given [51,59,61].

## Representative recruitment and equitable targeting of translation

The availability of healthcare, including access to the fruits of translation, is influential in attitudes to research participation, and these links need to be thought about carefully. Those who participate in research knowing that their healthcare is well provided for may well have different concerns to those individuals or groups whose healthcare needs are poorly served. Attitudes are markedly different in different countries and between different groups and it is vital for recruitment processes to understand this [69]. Groups with unmet health needs may feel reluctance to participate in research and this is especially the case where resource allocation is seen as unfair, as research amongst African Americans suggests [70]. It seems unlikely to be a coincidence that Swedes, who have generally good access to medical resources, often profess indifference to the sources of funding for research [51,71], whereas African Americans, many of whom lack health insurance and thus assured healthcare provision, have shown suspicion towards the motivations of researchers and their potential financial and personal rewards [70]. It is vital that robust levels of recruitment take place amongst certain underrepresented groups [72], especially as these are groups most likely to experience large burdens of ill health [69]; indeed, representative recruitment is now mandated in the US for reasons of equity as well as scientific integrity [73]. Researchers have limited control over large social issues, which require much broader efforts to improve equality and justice. However, forward planning about how the benefits of research translation may be fed back to participating groups and due care and attention to the recruitment process may assist in boosting participation rates as well as being a real contribution to health care justice. Ensuring that research directions and research agendas include attention to a full range of causal pathways, such as gene-environment interactions [22], may also in the long term help more effectively to address some of the reasons behind health disparities between different groups.

## Checklist for planning a translational research project in genomics

• Make sure appropriate and effective agreements are in place before commencement of research.

• Keep expectations of profits to reasonable levels, and do not let a desire for profit block translation of research into clinically useful innovations.

• Recognize and utilize the power of institutions and funding bodies to help ensure that innovations can be utilized for the maximum benefit to patients around the world.

• Address research participant concerns about commercialization, and ensure that research participants are fully informed through informed consent procedures about the potential commercial outcomes of research.

## Conclusions

Different clinical outcomes of genomics research will be best suited to different pathways of translation and development. There will be much greater investment in the development of a therapeutic, with its associated regulatory approval, than in recommendations of changes to lifestyle or medical treatment practices; private investment may be more appropriate and necessary in some areas than others. IP protection is likely to be important in the development of new innovations from genomics research. It should, however, be used sparingly and sensitively, in accordance with the best practices developed by organizations such as the OECD and the National Institutes of Health [34,35,41].

Translation need not only be about the generation of monopoly profits for the pharmaceutical industry. Ultimately, translation should be about ensuring that the benefits of genomics research reach those in the community who have the need and will benefit from them. When research institutions are planning genomics research, this goal should be kept in mind, and arrangements should be put in place to facilitate translation right from the beginning.

## Abbreviations

IP: intellectual property.

## Competing interests

The authors declare that they have no competing interests.

## Authors' contributions

All the authors have contributed to the preparation of this work.
